# Supplementary material for: A systematic study of key elements underlying molecular property prediction
Source: Nat Commun. 2023 Oct 13;14:6395. doi: 10.1038/s41467-023-41948-6 (PMC10575948; doi:10.1038/s41467-023-41948-6)
Supplement: Supplementary file 1 — Supplementary Information [file 41467_2023_41948_MOESM1_ESM.pdf]

Supplementary Information

Supplementary Table 1. Summary of fixed molecular representations

Type	Name	Dimension
Descriptors	RDKit2D	200
Descriptors	PhysChem	11
Structural Keys	MACCS	2048
Fingerprints	MorganBits	2048
Fingerprints	MorganCounts	2048
Fingerprints	AtomPairs	2048

Supplementary Table 2. Common node and edge features

Type	Feature	Notes
Node	Atom type	Element type
Node	Formal charge	Assigned charges
Node	Implicit Hs	Number of bonded hydrogens
Node	Chirality	*R* or *S* configuration
Node	Hybridization	Orbital hybridization
Node	Aromaticity	Aromatic atom or not
Edge	Bond type	Single, double, triple, aromatic
Edge	Conjugated	Conjugated or not
Edge	Stereoisomers	cis or trans (*E* or *Z*), none, any

Supplementary Table 3. Count of individual split where a model shows the best performance.

Dataset	BACE			BBBP			HIV		
Model	RF	MOLBERT	GROVER	RF	MOLBERT	GROVER	RF	MOLBERT	GROVER
AUROC	23	1	6	20	6	4	11	18	1
AUPRC	20	4	6	19	7	4	21	8	1
PPV	20	5	5	14	7	9	19	8	3
NPV	23	4	3	14	10	6	10	20	0
Dataset	ESOL			FreeSolv			Lipop		
Model	RF	MOLBERT	GROVER	RF	MOLBERT	GROVER	RF	MOLBERT	GROVER
RMSE	30	0	0	12	0	18	30	0	0
MAE	30	0	0	13	0	17	30	0	0
R2	30	0	0	12	0	18	30	0	0
PEARSON_R	30	0	0	10	0	20	29	0	1

Note[1]: prediction performance under scaffold split is used. Note[2]: fixed representation for RF is RDKit2D descriptors.
Note[3]: data are provided in the Source Data.

Supplementary Table 4. Count of triple-splits combinations where a model shows the best performance.

Dataset	BACE			BBBP			HIV		
Model	RF	MOLBERT	GROVER	RF	MOLBERT	GROVER	RF	MOLBERT	GROVER
AUROC	3,644	23	393	3,189	408	463	1,404	2,635	21
AUPRC	3,162	330	568	2,817	903	340	3,201	848	11
PPV	3,022	386	652	2,435	727	898	3,152	750	158
NPV	3,521	361	178	2,220	1,031	809	1,067	2,993	0
Dataset	ESOL			FreeSolv			Lipop		
Model	RF	MOLBERT	GROVER	RF	MOLBERT	GROVER	RF	MOLBERT	GROVER
RMSE	4,060	0	0	1,450	0	2,610	4,060	0	0
MAE	4,060	0	0	1,655	0	2,405	4,060	0	0
R2	4,060	0	0	1,506	0	2,554	4,060	0	0
PEARSON_R	4,060	0	0	912	0	3,148	4,060	0	0

Note[1]: prediction performance under scaffold split is used. Note[2]: fixed representation for RF is RDKit2D descriptors.
Note[3]: data are provided in the Source Data.

Supplementary Table 5. Summary of the MoleculeNet and opioids-related datasets.

Dataset	Task	#Molecule	Max. Len.	#Scaffold	Dataset	Task	#Molecule	Max. Len.	#Scaffold
BACE	CLS	1,513	198	737	MDR1	CLS/REG	1,438	252	602
BBBP	CLS	2,039	400	1,101	CYP2D6	CLS/REG	2,293	217	1,330
HIV	CLS	41,127	580	19,085	CYP3A4	CLS/REG	3,671	244	2,022
ESOL	REG	1,128	98	268	MOR	CLS/REG	3,553	373	1,623
FreeSolv	REG	642	82	62	DOR	CLS/REG	3,223	373	1,531
Lipop	REG	4,200	267	2,443	KOR	CLS/REG	3,326	373	1,660

Supplementary Table 6. Summary of commonly used statistical tests.

Statistical Test	Alias	Parametric	Normality	Equal Variance	Equal Size
Paired *t* test	Dependent *t* test	✓	✓	✓	✓
Unpaired *t* test	Independent or Welch's *t* test	✓	✓	×	×
Wilcoxon signed-rank test	-	×	×	✓	✓
Wilcoxon rank-sum test	Mann-Whitney *U* test	×	×	×	×

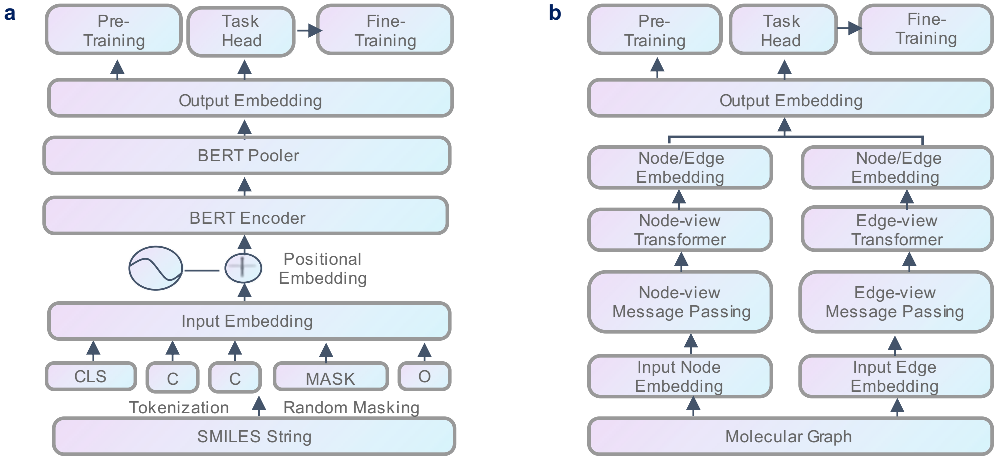

Supplementary Fig. 1. Abstracted model architectures for pretrained models. a. **MolBERT** An input SMILES string is tokenized and embedded into a sequence of d-dimensional vectors. Unlike RNNs, which process sequentially, a positional embedding layer is added to the input to capture the sequential information. Subsequently, a stack of n BERT encoder layers is added on top of the embedding layers to learn the latent representations of the input sequence. During pretraining, different pretext self-supervised tasks, such as masked language modeling, are designed to utilize the output embeddings after the pooler layer. During finetuning, new task heads can be appended by attaching a single linear layer to the pooled output for downstream prediction. The learned weights of the backbone model during pretraining can be fixed, which provide a better model initialization and reduce training burden in finetuning, especially for large models. **b**. **GROVER**. The input node and edge embeddings are first learned via message passing. These embeddings are then passed to the node-view transformer and edge-view transformer, respectively, to output the node and edge embeddings from both views. After a READOUT function, the final embeddings can be used for node-level, edge-level or graph-level prediction tasks.

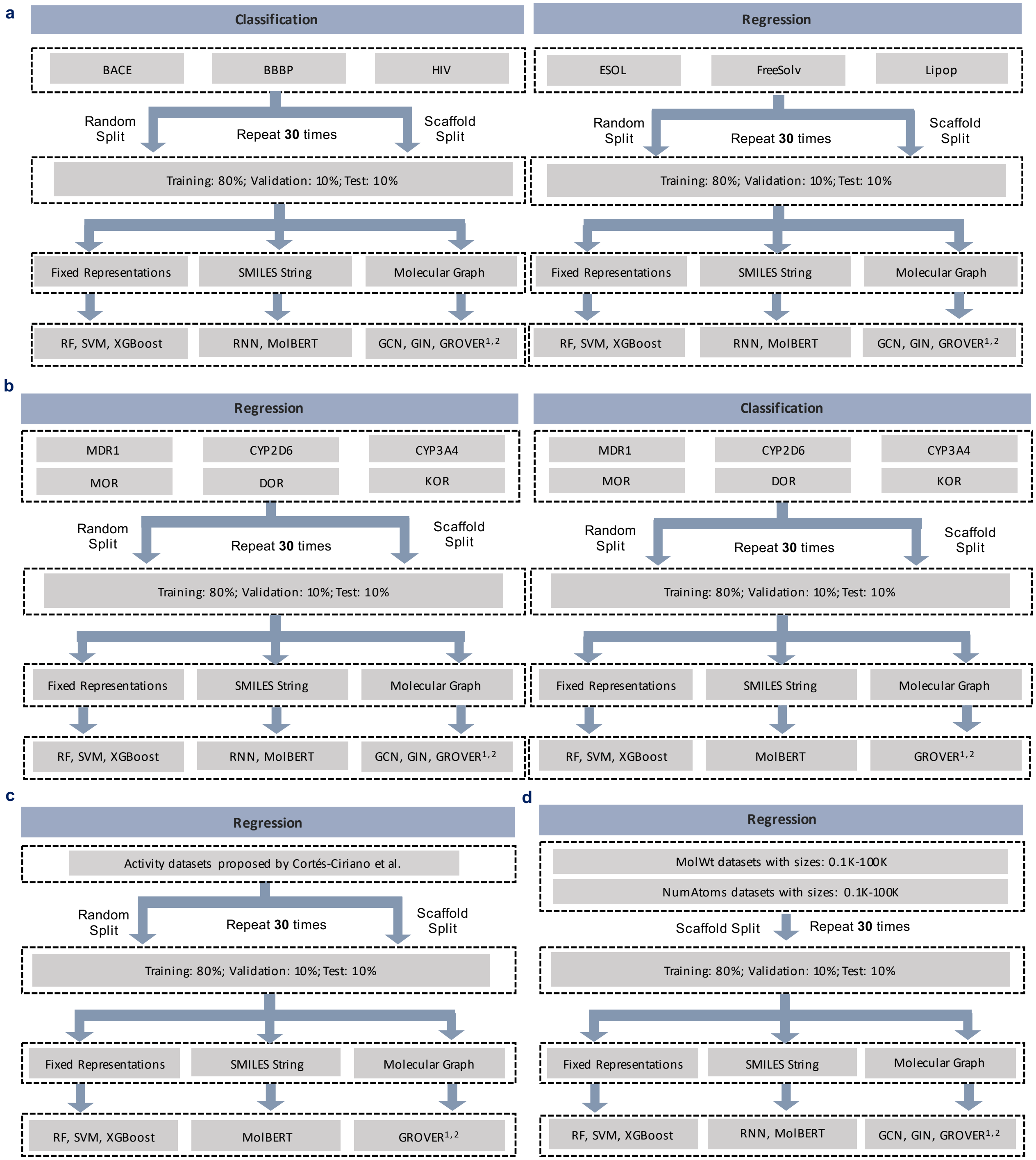

Supplementary Fig. 2. Experiment schemes on various sets of datasets. a. Evaluation using MoleculeNet datasets at regression and classification settings. **b**. Evaluation using opioids-related datasets at regression and classification settings. **c**. Evaluation using activity datasets by Cortés-Ciriano *et al.*at regression setting. **d**. Evaluation using descriptor (MolWt, NumAtoms) datasets at regression setting.

Note[1]: for these datasets, we split them into training, validation and test sets, which are kept consistent for each representation-model combination. Note[2]: for activity datasets by Tilborg *et al.*, data split is fixed so we just adopted its spit and only applied RF, SVM, XGBoost on fixed representations. Note[3]: GROVER[1,2] stands for GROVER and GROVER_RDKit, respectively. Note[4]: fixed representations include RDKit2D descriptors, PhysChem descriptors, MorganBits fingerprints, MorganCounts fingerprints, MACCS keys and AtomPairs fingerprints.

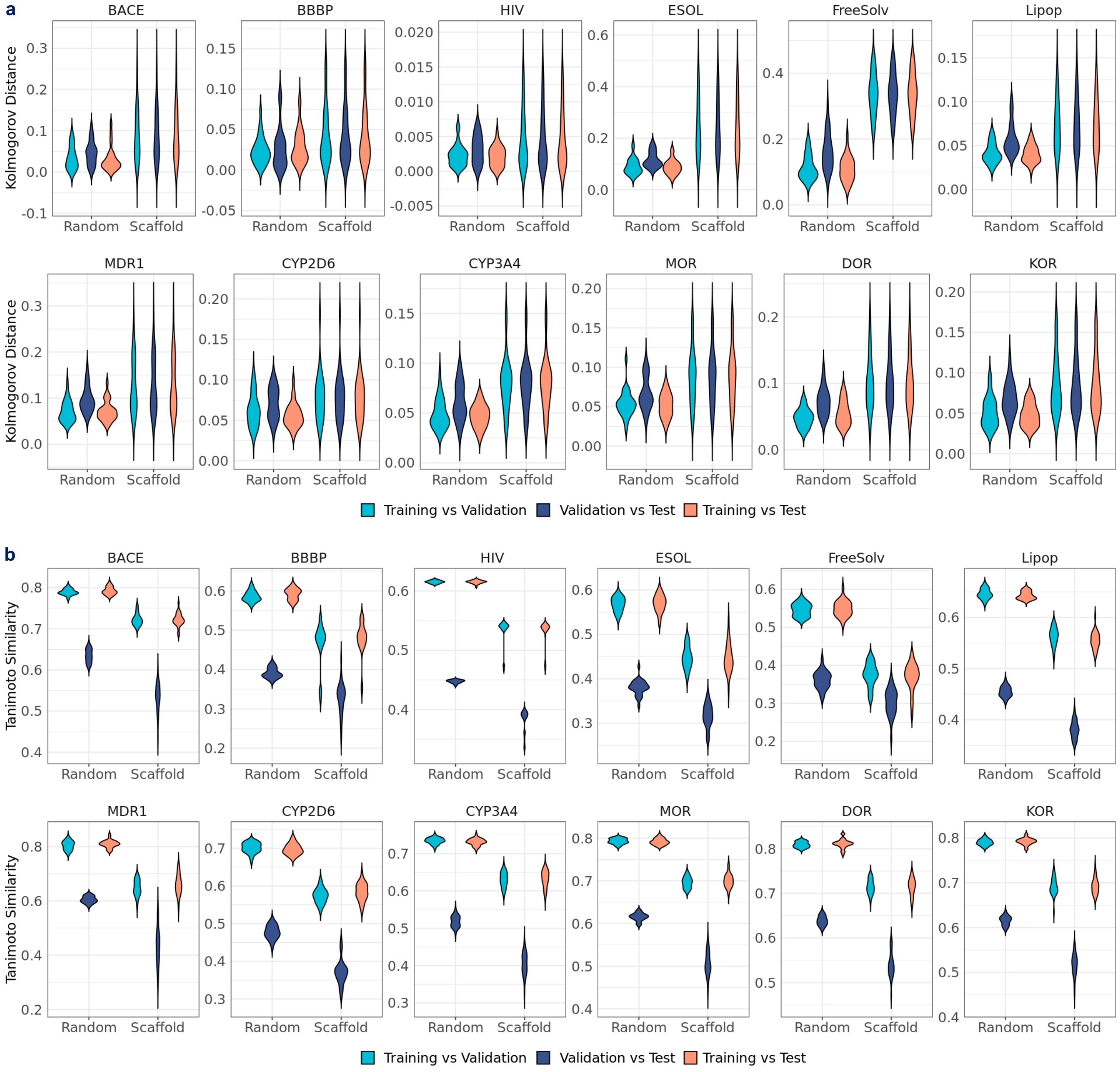

Supplementary Fig. 3. Distribution of label divergence and structure similarity in the MoleculeNet datasets and opioids-related datasets over 30 splits. a. Distribution of Kolmogorov distance among training, validation, and test sets. **b**. Distribution of Tanimoto similarity among training, validation, and test sets.

Note[1]: data are in the Source Data.

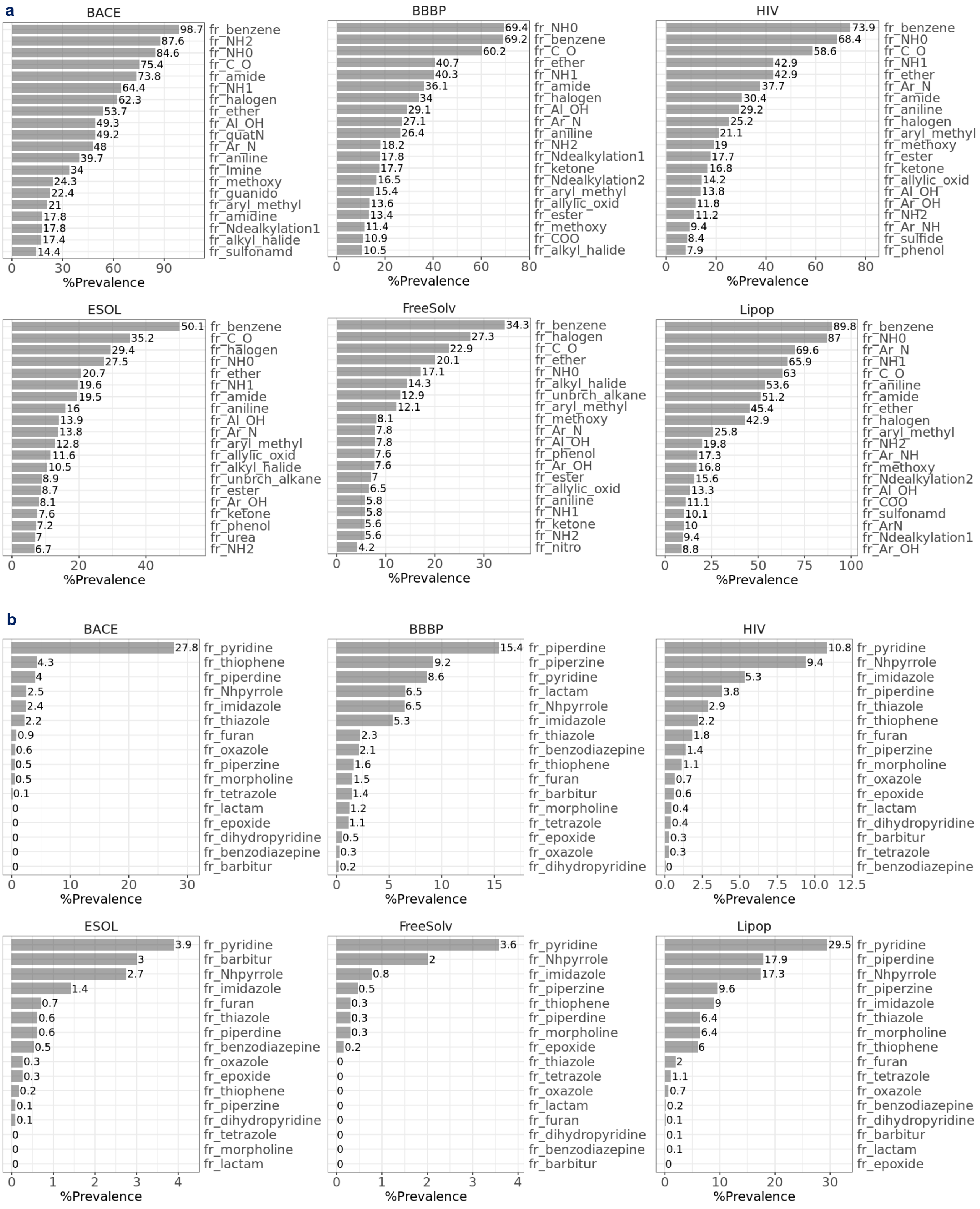

Supplementary Fig. 4. Top fragments prevalence in the MoleculeNet datasets. a. Prevalence of top heterocycles. **b**. Prevalence of top heterocycles functional groups.

Note[1]: data are provided in the Source Data.

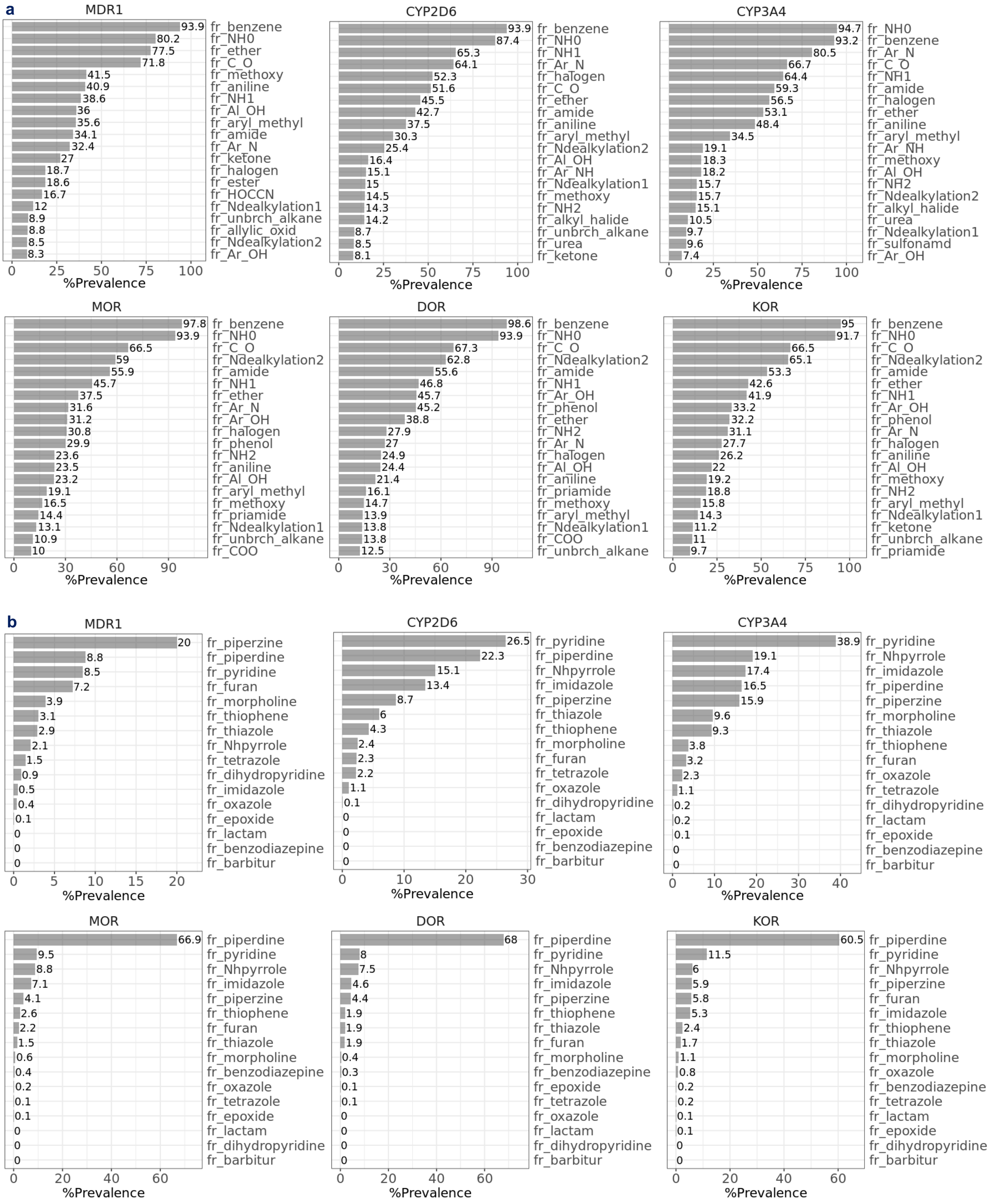

Supplementary Fig. 5. Top fragments prevalence in the opioids-related datasets. **a**. Prevalence of top heterocycles. **b**. Prevalence of top heterocycles functional groups.
Note[1]: data are in the Source Data.

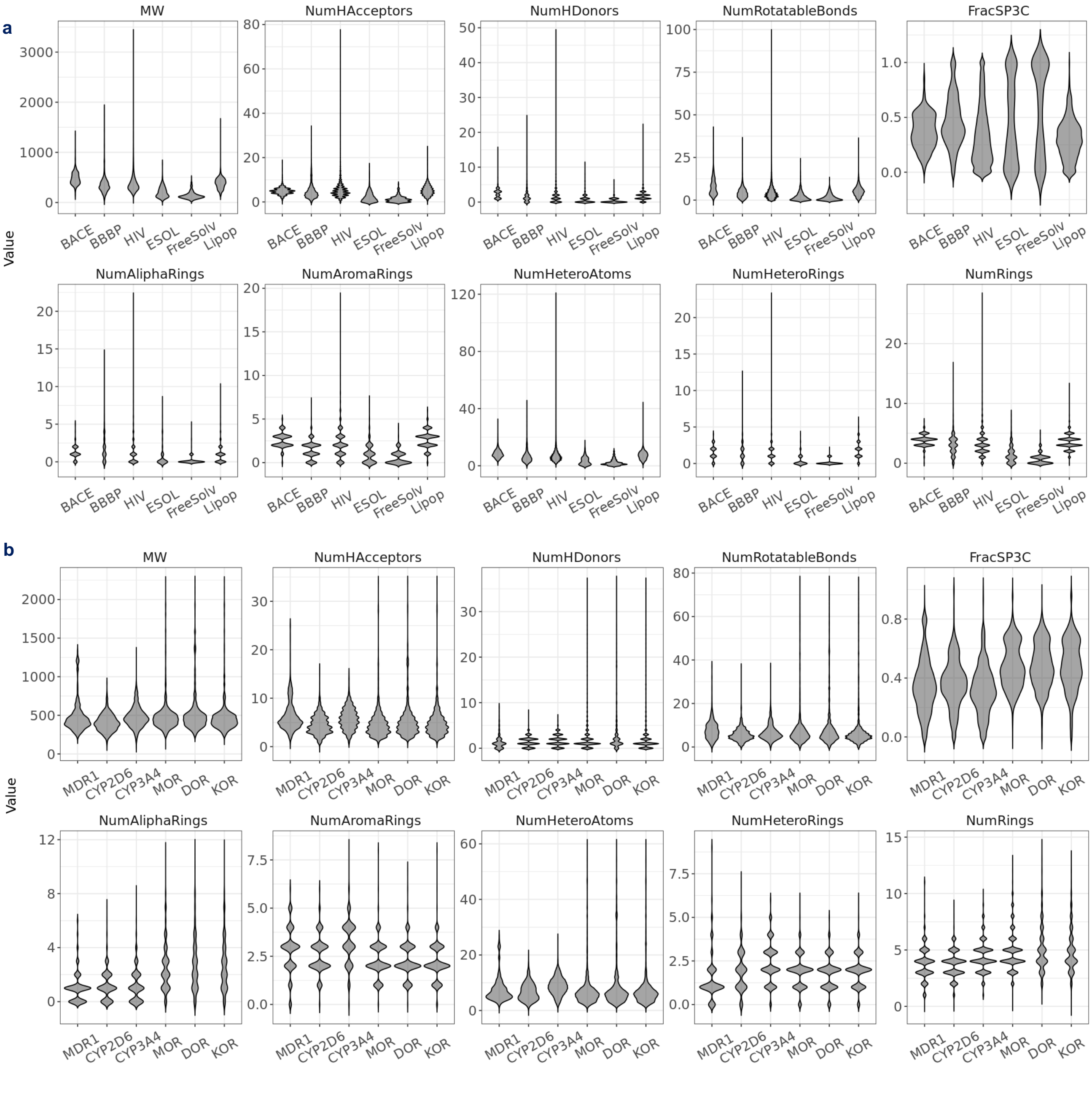

Supplementary Fig. 6. **Examining distribution of other structural traits. a**. Violin plot for structural traits values in the MoleculeNet datasets. **b**. Violin plot for structural traits values in the opioids-related datasets.
Note[1]: data are in the Source Data.

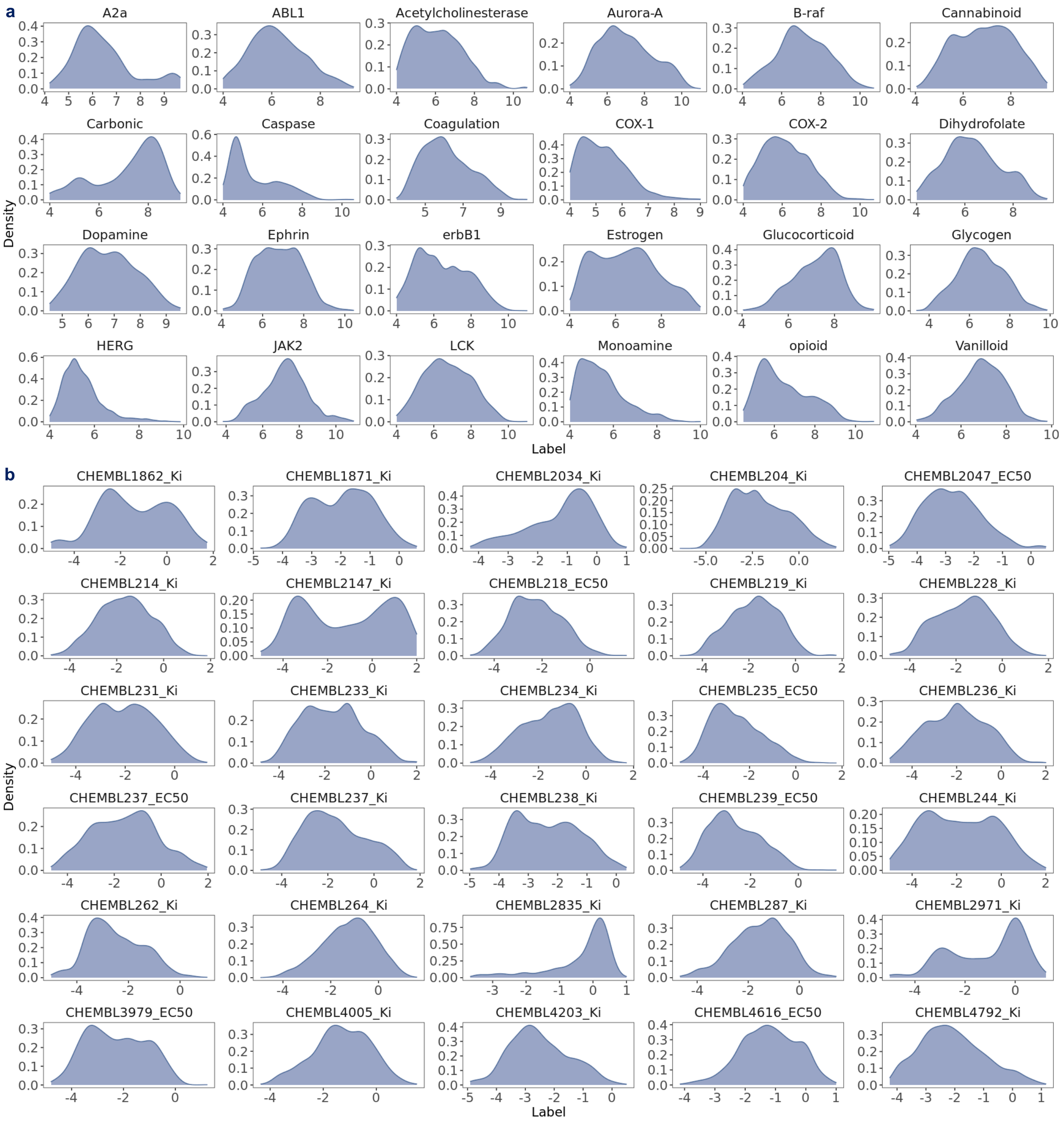

Supplementary Fig. 7. Label distribution in the activity datasets. a. Activity distribution for 24 targets in the datasets by Cortés-Ciriano *et al*.**b**. Activity distribution for 30 targets in the datasets by Tilborg *et al*.
Note[1]: data are in the Source Data.

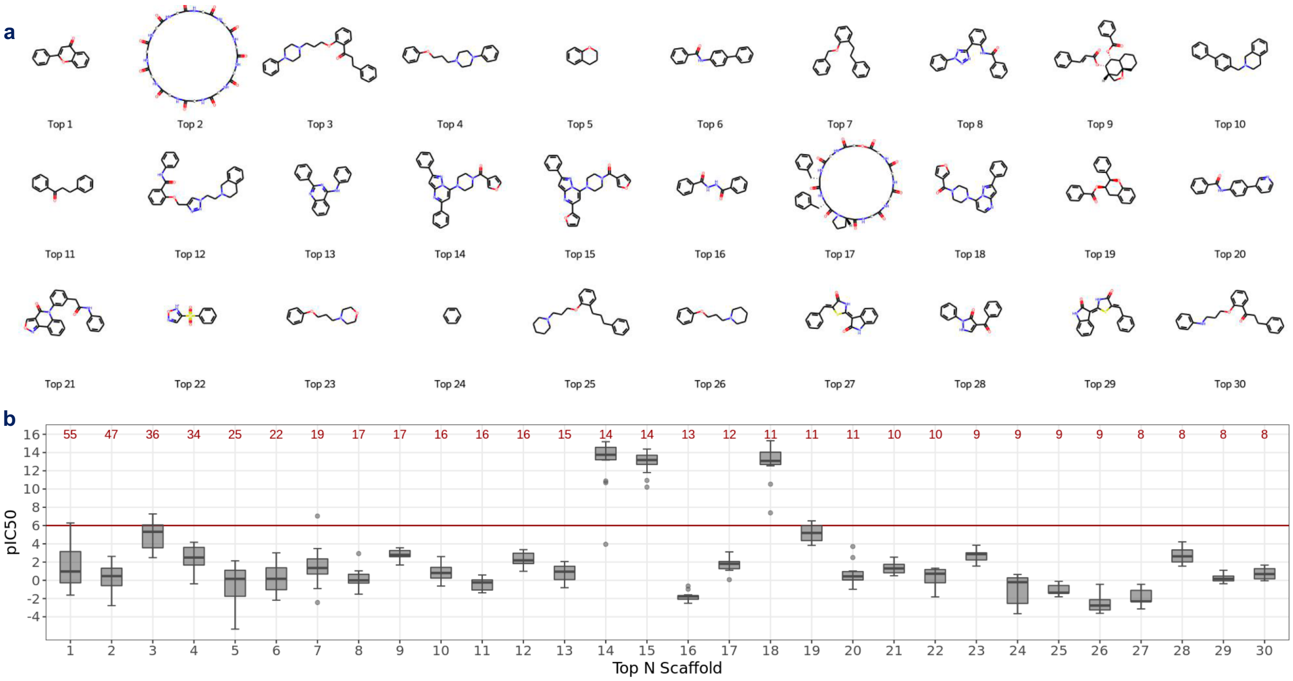

Supplementary Fig. 8. **Examining scaffolds and associated binding activity distribution in MDR1.** **a**. Top 30 scaffolds visualization. **b**. pIC50 distribution for molecules with top scaffolds. Note[1]: pIC50 is the negative logarithm of half maximal inhibitory concentration. Note[2]: red number is the count of molecules with top *N* scaffold. Note[3]: red line is the activity cutoff at 6. Note[4]: center line in the box plots denote the median; limits denote lower and upper quartiles; whiskers denote the range within 1.5 times interquartile from the median; points are outliers. Note[5]: data are in the Source Data.

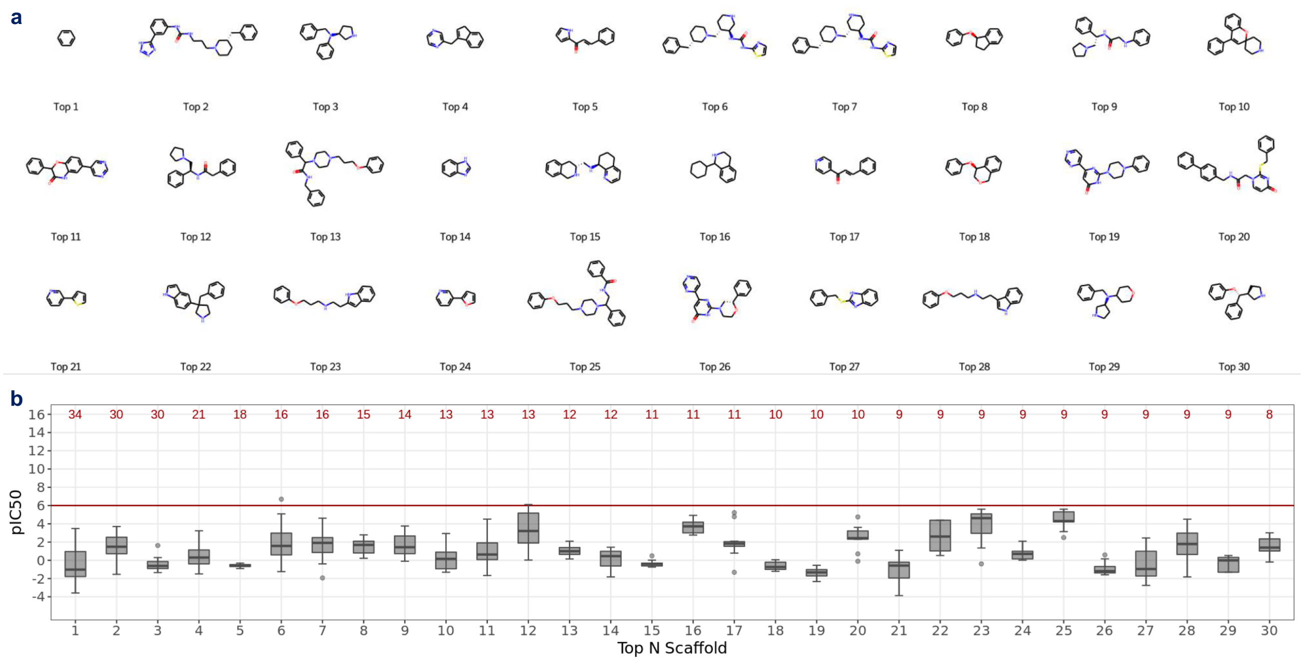

Supplementary Fig. 9. **Examining top scaffolds and associated binding activity distribution in CYP2D6.** **a**. Top 30 scaffolds visualization. **b**. pIC50 distribution for molecules with top scaffolds. Note[1]: pIC50 is the negative logarithm of half maximal inhibitory concentration. Note[2]: red number is the count of molecules with top *N* scaffold. Note[3]: red line is the activity cutoff at 6. Note[4]: center line in the box plots denote the median; limits denote lower and upper quartiles; whiskers denote the range within 1.5 times interquartile from the median; points are outliers. Note[5]: data are in the Source Data.

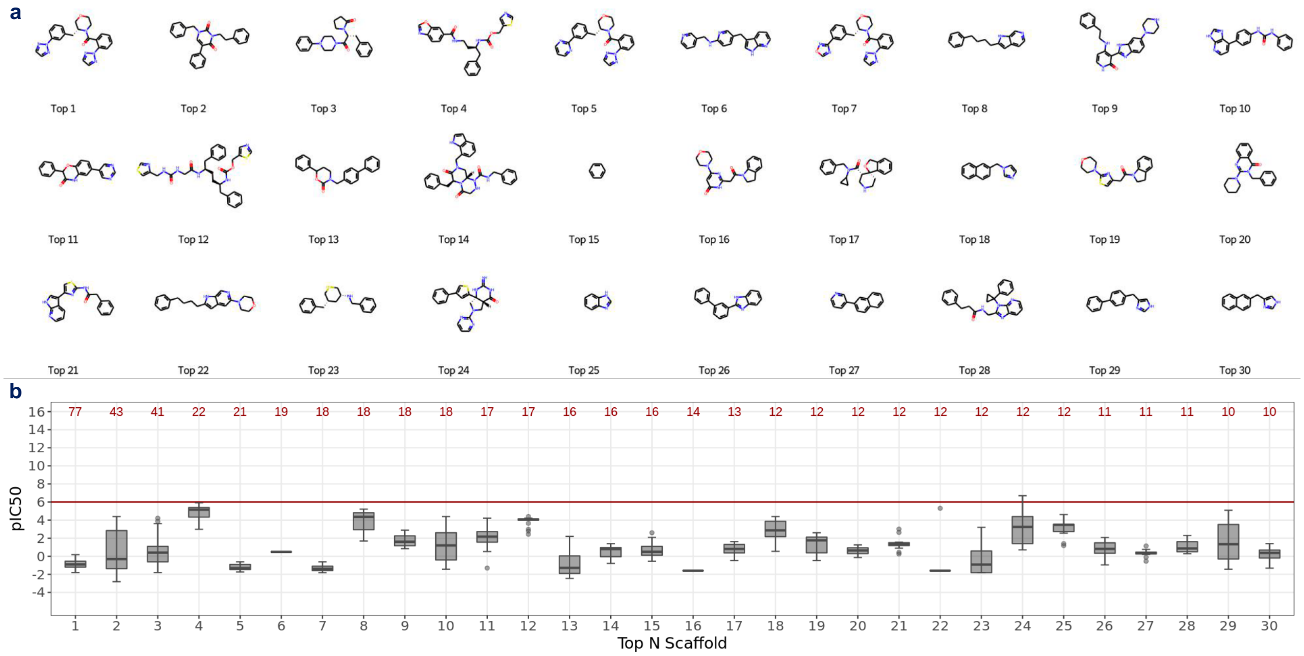

Supplementary Fig. 10. Examining top scaffolds and associated binding activity distribution in CYP3A4. a. Top 30 scaffolds visualization. **b**. pIC50 distribution for molecules with top scaffolds. Note[1]: pIC50 is the negative logarithm of half maximal inhibitory concentration. Note[2]: red number is the count of molecules with top *N* scaffold. Note[3]: red line is the activity cutoff at 6. Note[4]: center line in the box plots denote the median; limits denote lower and upper quartiles; whiskers denote the range within 1.5 times interquartile from the median; points are outliers. Note[5]: data are in the Source Data.

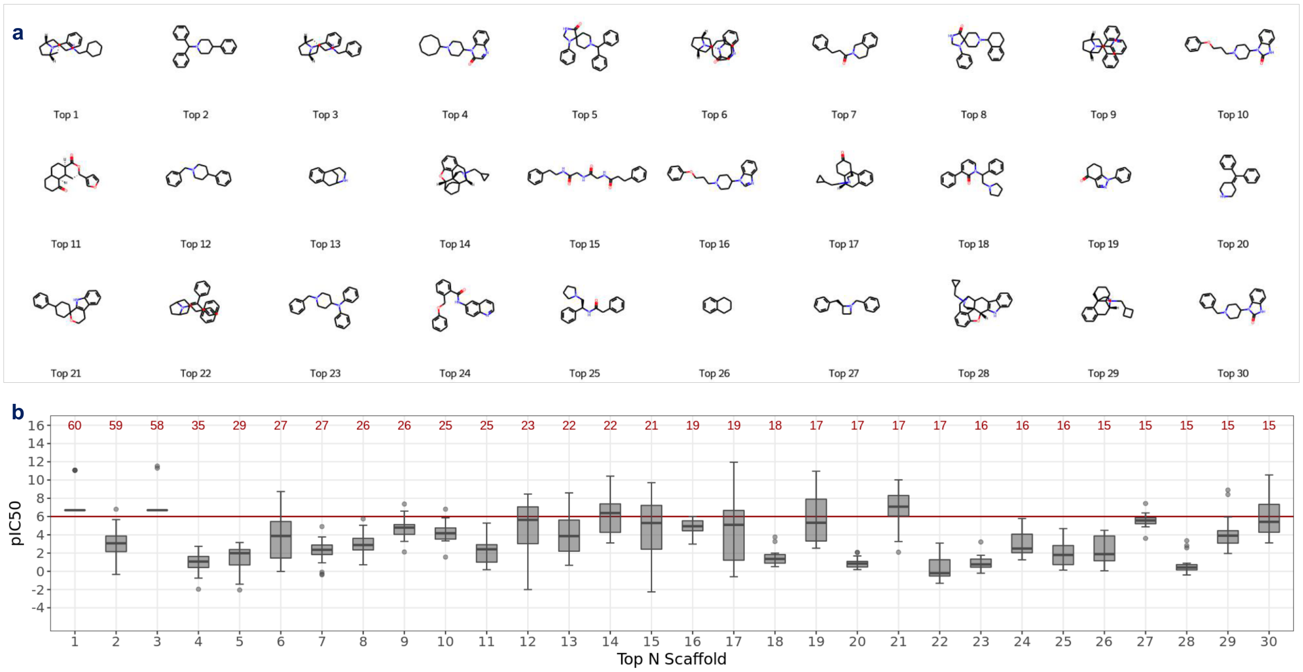

Supplementary Fig. 11. Examining top scaffolds and associated binding activity distribution in MOR. a. Top 30 scaffolds visualization. **b**. pIC50 distribution for molecules with top scaffolds. Note[1]: pIC50 is the negative logarithm of half maximal inhibitory concentration. Note[2]: red number is the count of molecules with top *N* scaffold. Note[3]: red line is the activity cutoff at 6. Note[4]: center line in the box plots denote the median; limits denote lower and upper quartiles; whiskers denote the range within 1.5 times interquartile from the median; points are outliers. Note[5]: data are in the Source Data.

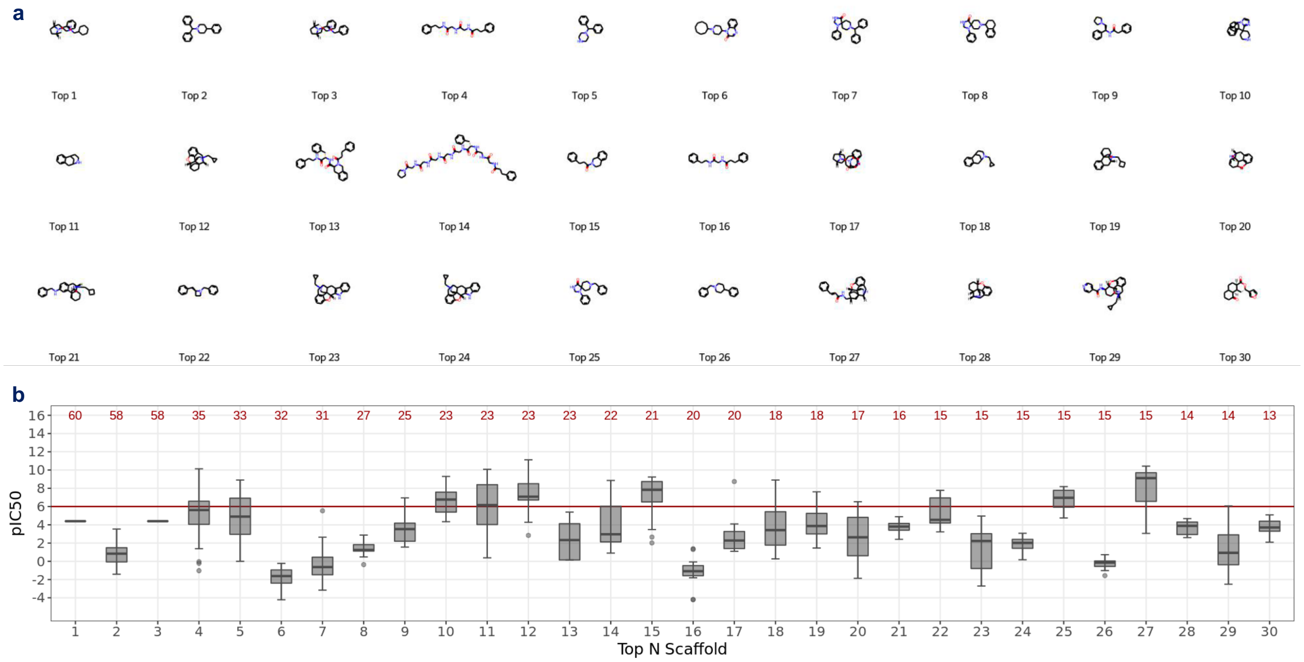

Supplementary Fig. 12. Examining top scaffolds and associated binding activity distribution in DOR. **a**. Top 30 scaffolds visualization. **b**. pIC50 distribution for molecules with top scaffolds. Note[1]: pIC50 is the negative logarithm of half maximal inhibitory concentration. Note[2]: red number is the count of molecules with top *N* scaffold. Note[3]: red line is the activity cutoff at 6. Note[4]: center line in the box plots denote the median; limits denote lower and upper quartiles; whiskers denote the range within 1.5 times interquartile from the median; points are outliers. Note[5]: data are in the Source Data.

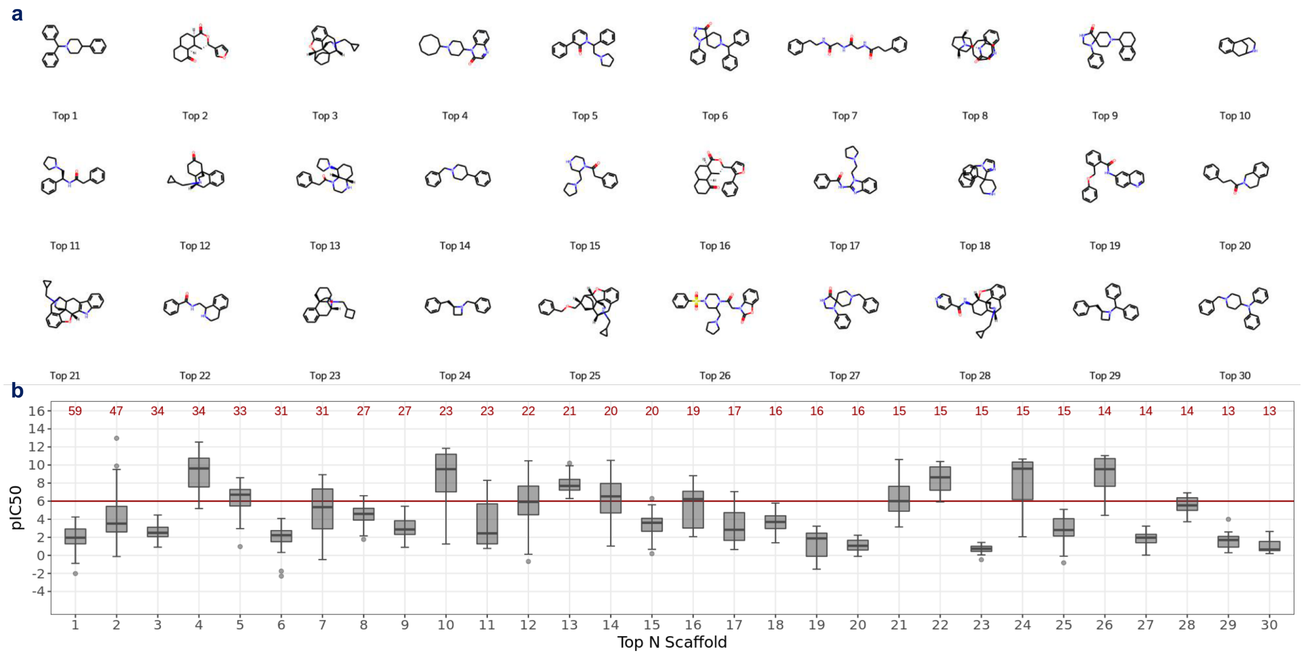

Supplementary Fig. 13. Examining top scaffolds and associated binding activity distribution in KOR. **a**. Top 30 scaffolds visualization. **b**. pIC50 distribution for molecules with top scaffolds. Note[1]: pIC50 is the negative logarithm of half maximal inhibitory concentration. Note[2]: red number is the count of molecules with top *N* scaffold. Note[3]: red line is the activity cutoff at 6. Note[4]: center line in the box plots denote the median; limits denote lower and upper quartiles; whiskers denote the range within 1.5 times interquartile from the median; points are outliers. Note[5]: data are in the Source Data.

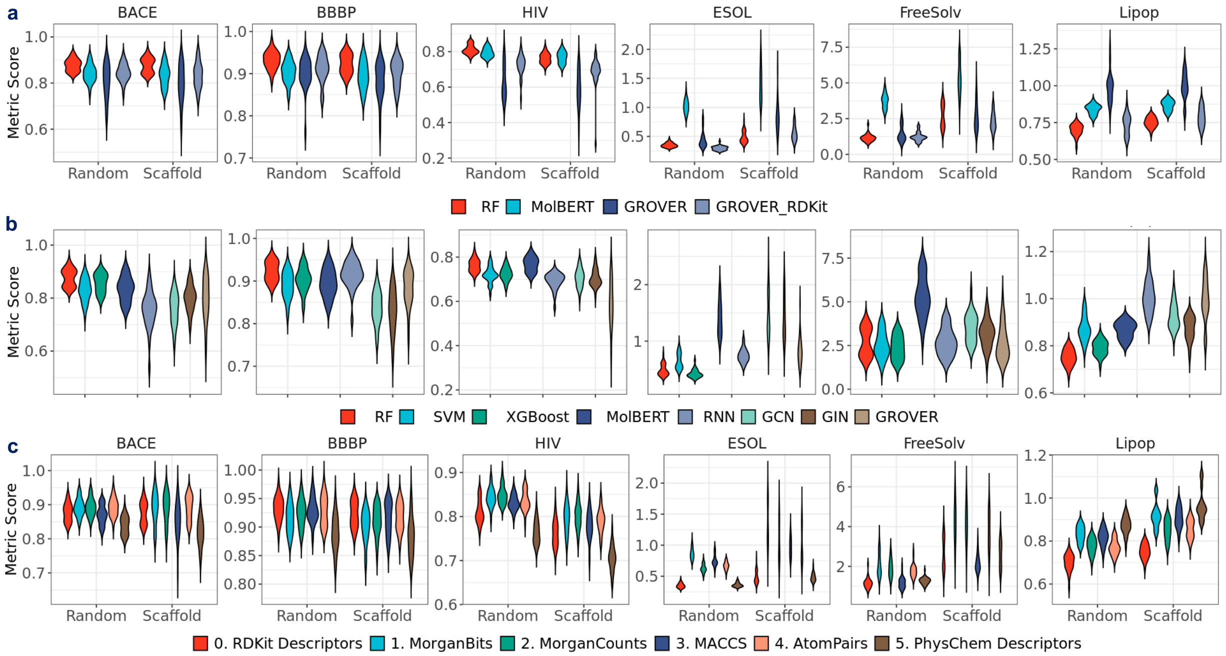

Supplementary Fig. 14. Examining performance metric distribution in the MoleculeNet datasets. a. Violin plot for RF on RDKit2D descriptors, MolBERT, GROVER and GROVER_RDKit using default metrics. **b**. Violin plot for RF, SVM, and XGBoost on RDKit2D descriptors, RNN, and MolBERT, and GCN, GIN, and GROVER under scaffold split using default metrics. **c**. Violin plot for RF on different fixed representations using default metrics. Note[1]: default metric for classification datasets (BACE, BBBP, HIV) is AUROC and RMSE for regression datasets (ESOL, FreeSolv, Lipop). Note[2]: AUROC stands for Area Under the Receiver Operating Characteristic Curve; RMSE stands for Root Mean Square Error. Note[3]: data are in the Source Data.

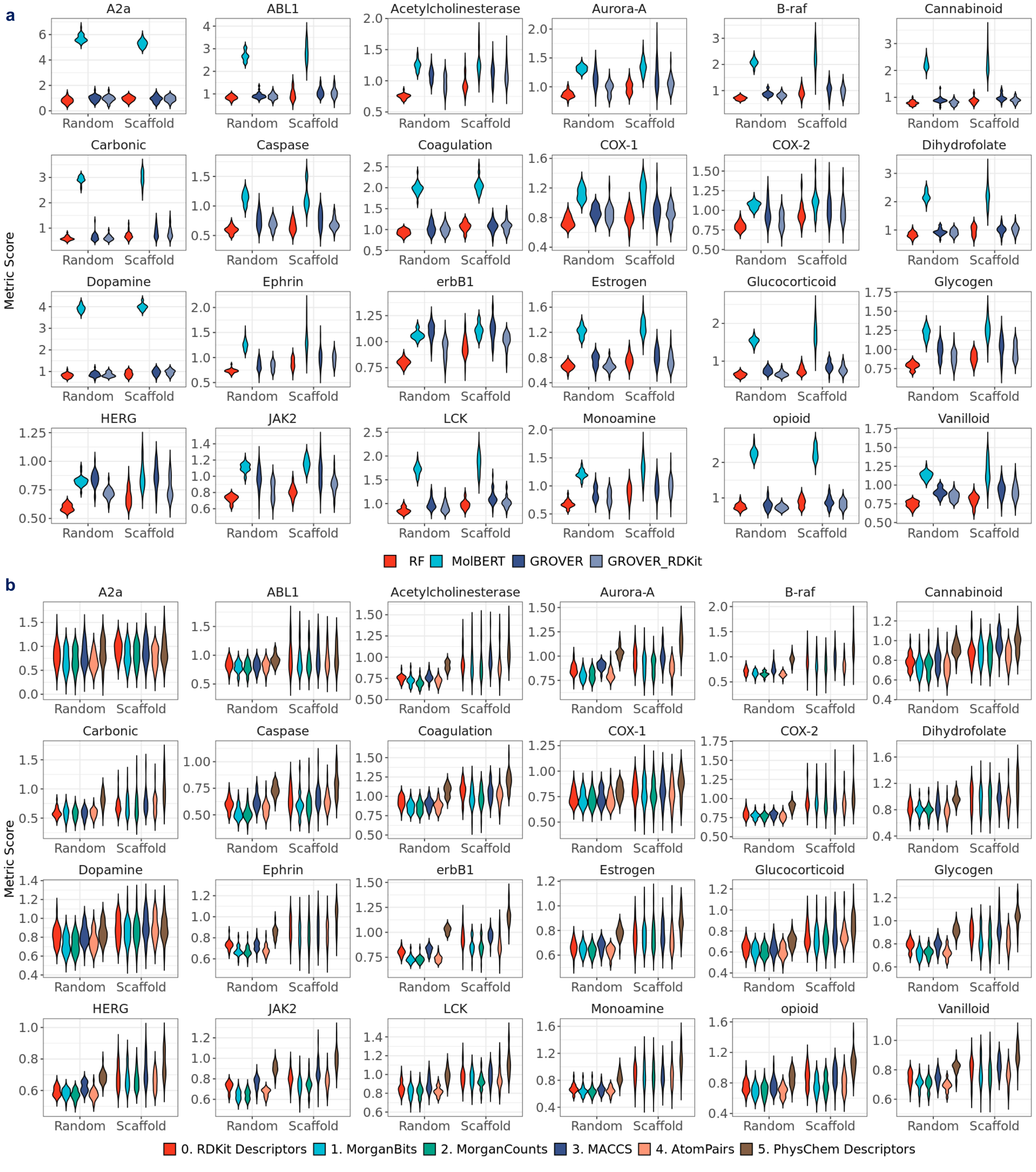

Supplementary Fig. 15. Examining performance metric distribution in the activity datasets by Cortés-Ciriano *et al*.. **a**. Violin plot for RF on RDKit2D descriptors, MolBERT, GROVER and GROVER_RDKit. **b**. Violin plot for RF on different fixed representations. Note[1]: default metric is RMSE. Note[2]: RMSE stands for Root Mean Square Error. Note[3]: data are in the Source Data.

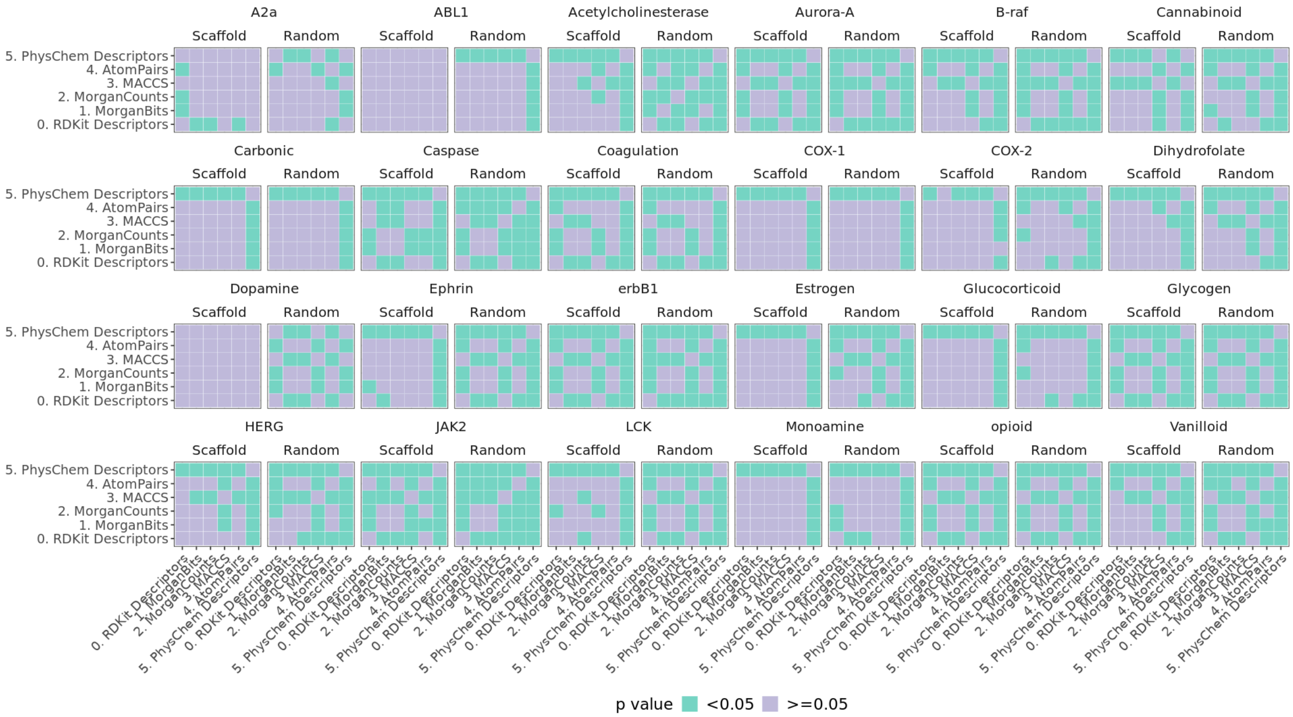

Supplementary Fig. 16. **Examining statistical significance for pairwise fixed representation comparison with activity datasets by Cortés-Ciriano *et al*.**
Note[1]: significance hotmap for pairwise representation comparison is a supplement for Fig. 4**c** in the main text. Note[2]: data are in the Source Data.

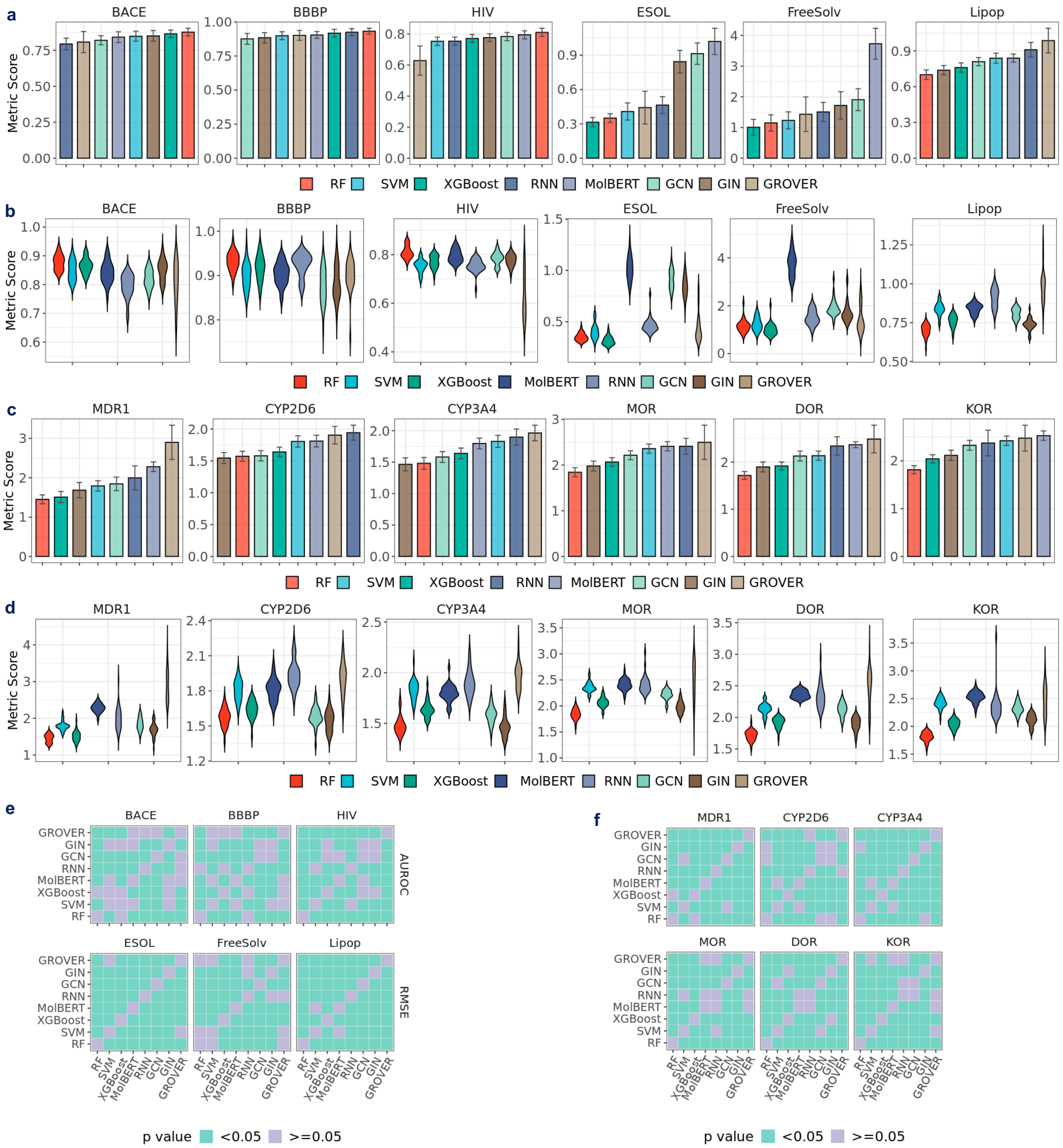

Supplementary Fig. 17. Evaluating prediction performance under random split. a. Prediction performance of RF, SVM, and XGBoost on RDKit2D descriptors, RNN, and MolBERT, and GCN, GIN, and GROVER with MoleculeNet datasets using default metrics. **b**. Violin plot for prediction performance (RMSE) of RF, SVM, and XGBoost on RDKit2D descriptors, RNN, and MolBERT, and GCN, GIN, and GROVER with MoleculeNet datasets. **c**. Prediction performance (RMSE) of RF, SVM, and XGBoost on RDKit2D descriptors, RNN, and MolBERT, and GCN, GIN, and GROVER with opioids-related datasets at regression setting. **d**. Violin plot for prediction performance (RMSE) of RF, SVM & XGBoost on RDKit2D descriptors, RNN & MolBERT, and GCN, GIN & GROVER with opioids-related datasets. **e**. Statistical significance for pairwise model comparison in **a**. **f**. Statistical significance for pairwise model comparison in **c**.

Note[1]: default metric for classification datasets (BACE, BBBP, HIV) is AUROC and RMSE for regression datasets (ESOL, FreeSolv, Lipop). Note[2]: error bar denotes standard deviation over 30 splits. Note[3]: Mann-Whitney U test is used for statistical analysis. Note[4]: AUROC stands for area under the receiver operating characteristic curve; RMSE stands for root mean square error. Note[5]: data are in the Source Data.

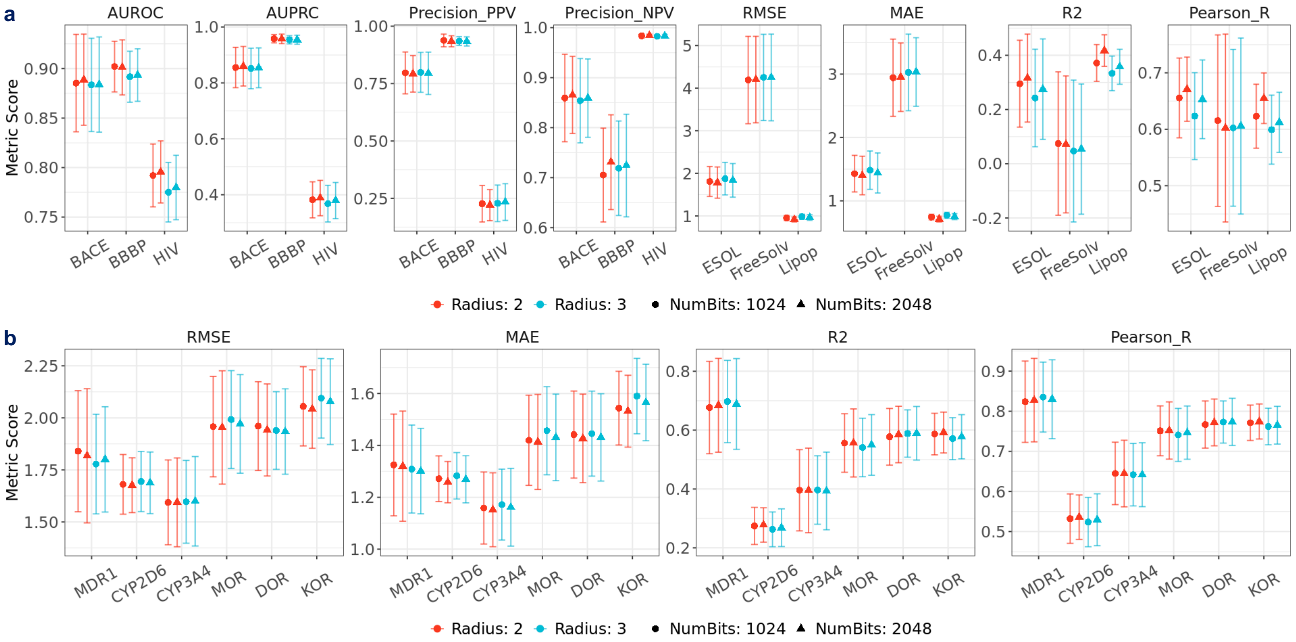

Supplementary Fig. 18. Evaluating prediction performance using RF on MorganBits fingerprints under scaffold split. a. Prediction performance of RF on MorganBits fingerprints with MoleculeNet datasets. **b**. Prediction performance of RF on MorganBits fingerprints with opioids-related datasets at regression setting.
Note[1]: error bar denotes standard deviation over 30 splits. Note[2]: statistically significant difference (Radius 2 vs 3) in HIV (NumBits: 1024; AUROC) and Lipop (NumBits: 1024, 2048; RMSE, MAE, R2, Pearson_R) . Note[3]: AUROC is area under the receiver operating characteristic curve; AUPRC is area under the precision recall curve; Precision_PPV is a precision metric for positive predictive value; Precision_NPV is a precision metric for negative predictive value. Note[4]: RMSE is root mean square error; MAE is mean absolute error; R2 is coefficient of determination; Pearson_R is Pearson correlation coefficient. Note[5]: data are in the Source Data.

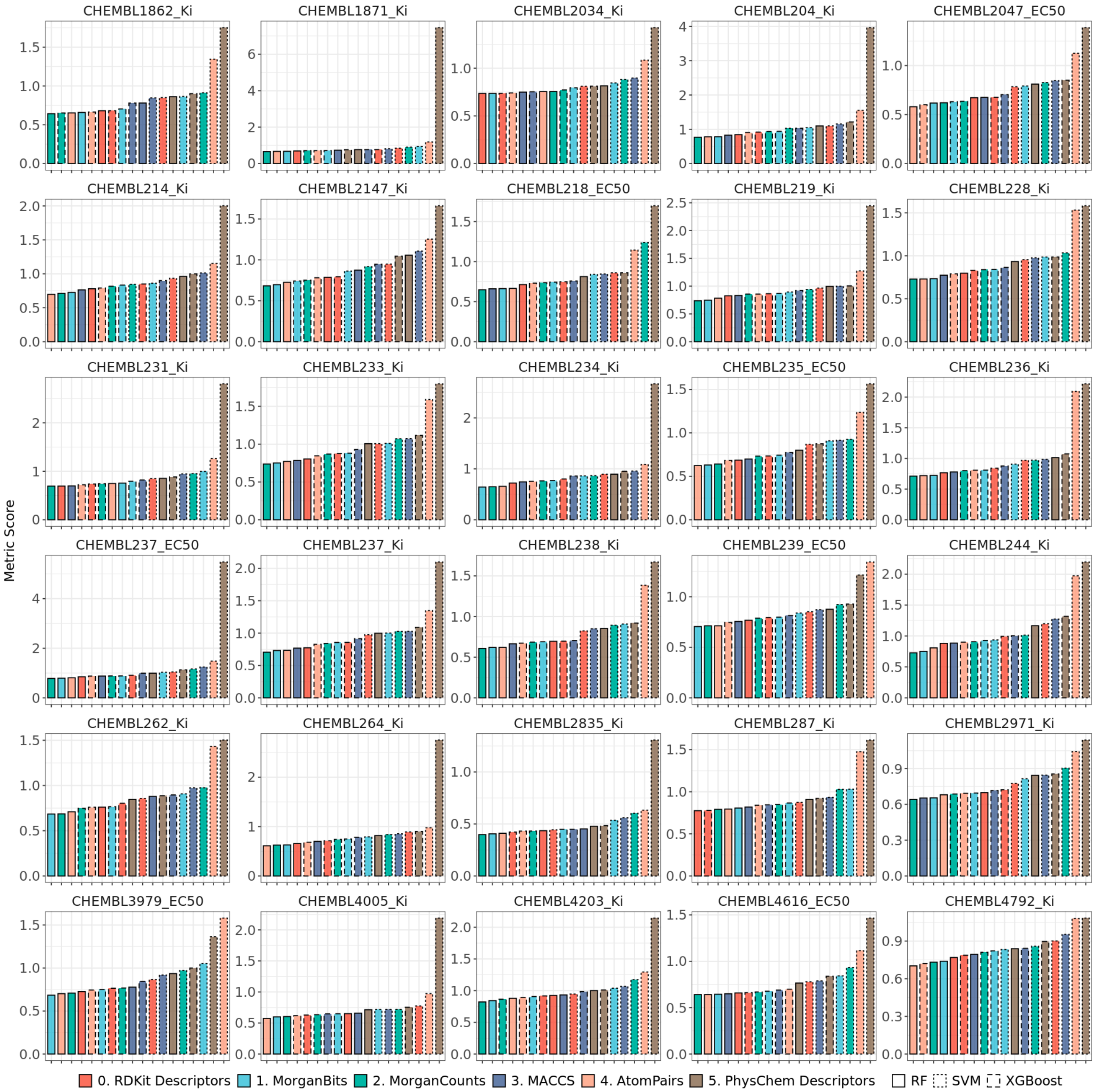

Supplementary Fig. 19. **Evaluating prediction performance with activity datasets by Tilborg *et al*.**
Note[1]: traditional machine learning models RF, SVM, XGBoost are applied on different fixed representations. Note[2]: evaluation metric is RMSE. Note[3]: RMSE stands for Root Mean Square Error. Note[4]: data are in the Source Data.

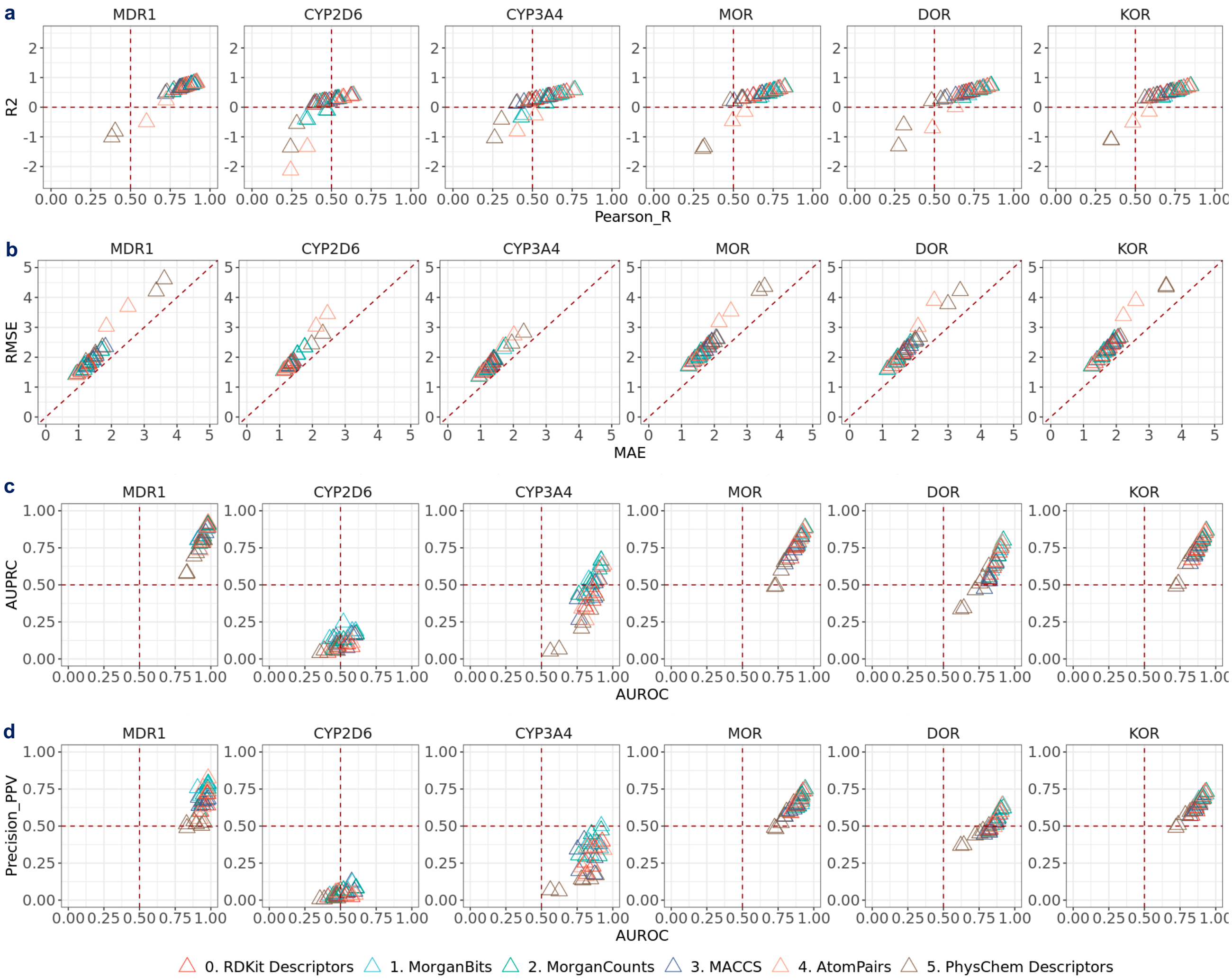

Supplementary Fig. 20. Examining metrics relationship with opioids-related datasets. a. Relationship between R2 and Pearson_R. **b**. Relationship between RMSE and MAE. **c**. Relationship between AUPRC and AUROC. **d**. Relationship between Precision_PPV and AUROC.

Note[1]: prediction results are based on RF on fixed representations. Note[2]: red dashed lines in **a** denote the boundary lines where R2 is 0 and Pearson_R is 0.5. Note[3]: red dashed line in **b** denote the $y = x$ line. Note[4]: red dashed lines in **a** denote the boundary lines where AUROC is 0.5 and AUPRC is 0.5. Note[5]: red dashed lines in **a** denote the boundary lines where AUROC is 0.5 and Precision_PPV is 0.5. Note[6]: R2 is coefficient of determination; Pearson_R is Pearson correlation coefficient; RMSE is root mean square error; MAE is mean absolute error. Note[7]: AUROC is area under the receiver operating characteristic curve; AUPRC is area under the precision recall curve; Precision_PPV is a precision metric for positive predictive value. Note[8]: data are in the Source Data.

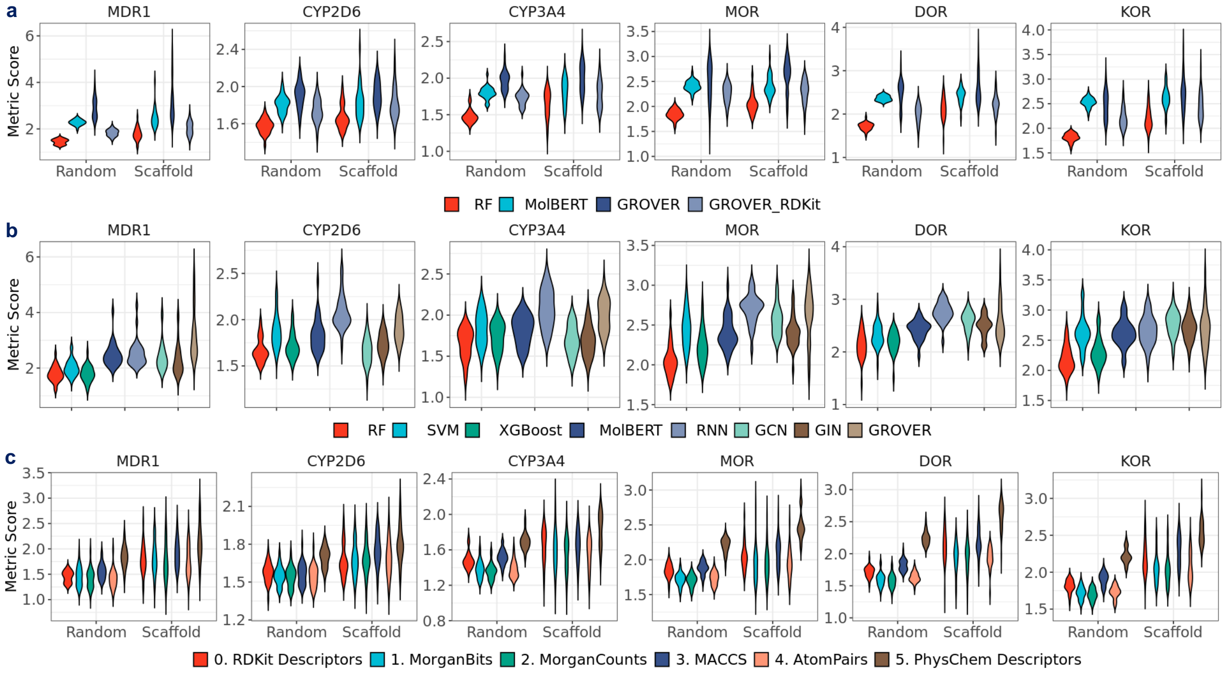

Supplementary Fig. 21. Examining performance metric distribution in the opioids-related datasets at regression setting. a. Violin plot for RF on RDKit2D descriptors, MolBERT, GROVER and GROVER_RDKit. **b**. Violin plot for RF, SVM, and XGBoost on RDKit2D descriptors, RNN, and MolBERT, and GCN, GIN, and GROVER under scaffold split. **c**. Violin plot for RF on different fixed representations.
Note[1]: default metric is RMSE. Note[2]: RMSE stands for Root Mean Square Error. Note[3]: data are in the Source Data.

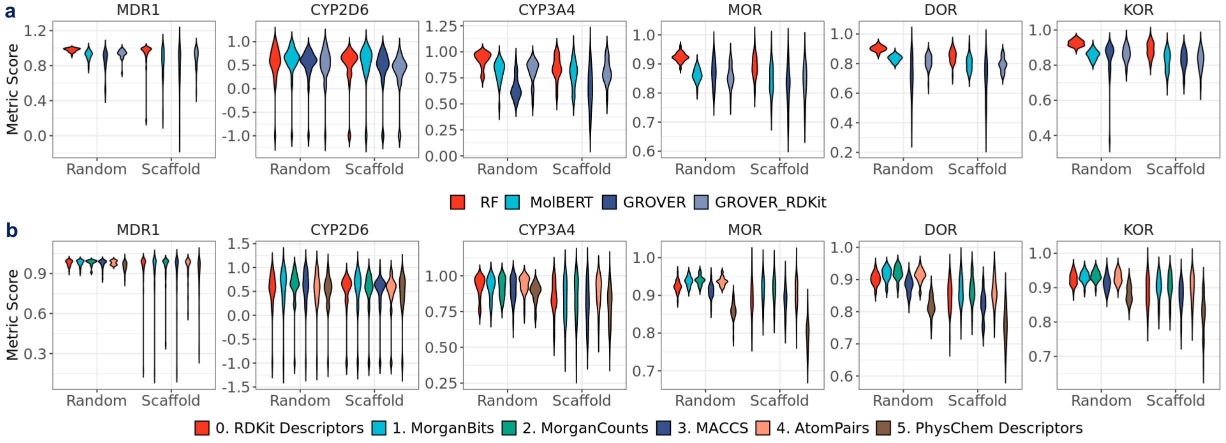

Supplementary Fig. 22. Examining performance metric distribution in the opioids-related datasets at classification setting. a. Violin plot for RF on RDKit2D descriptors, MolBERT, GROVER and GROVER_RDKit. **b**. Violin plot for RF on various fixed representations. Note[1]: default metric is AUROC. Note[2]: AUROC stands for Area Under the Receiver Operating Characteristic Curve. Note[3]: data are in the Source Data.

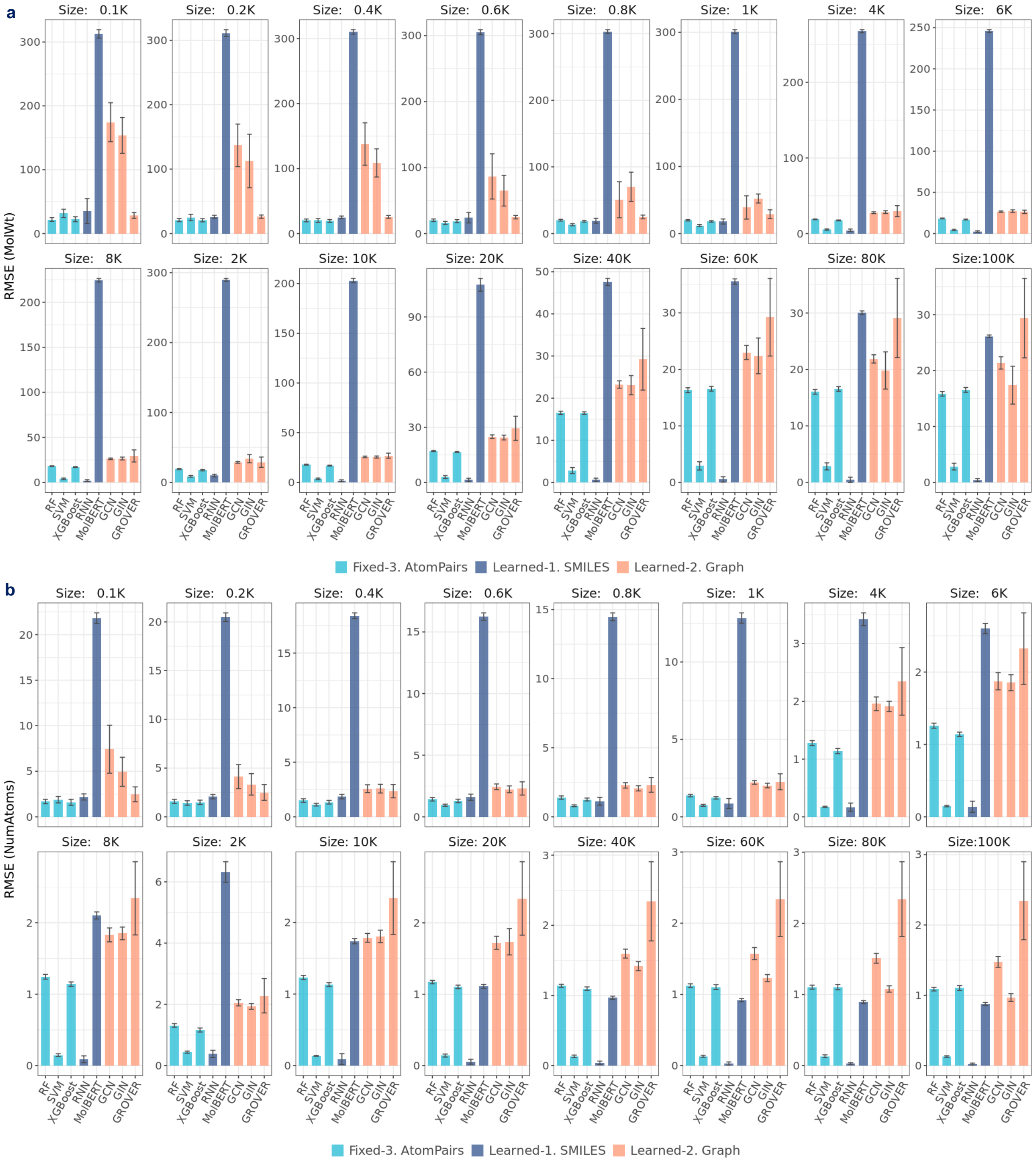

Supplementary Fig. 23. **Comparing prediction performance at different dataset sizes a**. Prediction performance (RMSE) of RF, SVM & XGBoost on AtomPairs fingerprints, RNN & MolBERT, and GCN, GIN & GROVER with MolWt datasets. **b**. Prediction performance (RMSE) of RF, SVM & XGBoost on AtomPairs fingerprints, RNN & MolBERT, and GCN, GIN & GROVER with NumAtoms datasets.
Note[1]: RMSE stands for Root Mean Square Error. Note[2]: data are in the Source Data.
